# Secure Cloud-Based Solutions for Different eHealth Services in Spanish Rural Health Centers

**DOI:** 10.2196/jmir.4422

**Published:** 2015-07-27

**Authors:** Isabel de la Torre- Díez, Miguel Lopez-Coronado, Begonya Garcia-Zapirain Soto, Amaia Mendez-Zorrilla

**Affiliations:** ^1^ Grupo de Telemedicina y eSalud Departamento de Teoría de la Señal y Comunicaciones e Ingeniería Telemática University of Valladolid Valladolid Spain; ^2^ DeustoTech Institute of Technology DeustoTech-Life Lab University of Deusto Bilbao Spain

**Keywords:** cloud, eHealth services, rural, security

## Abstract

**Background:**

The combination of eHealth applications and/or services with cloud technology provides health care staff—with sufficient mobility and accessibility for them—to be able to transparently check any data they may need without having to worry about its physical location.

**Objective:**

The main aim of this paper is to put forward secure cloud-based solutions for a range of eHealth services such as electronic health records (EHRs), telecardiology, teleconsultation, and telediagnosis.

**Methods:**

The scenario chosen for introducing the services is a set of four rural health centers located within the same Spanish region. iCanCloud software was used to perform simulations in the proposed scenario. We chose online traffic and the cost per unit in terms of time as the parameters for choosing the secure solution on the most optimum cloud for each service.

**Results:**

We suggest that load balancers always be fitted for all solutions in communication together with several Internet service providers and that smartcards be used to maintain identity to an appropriate extent. The solutions offered via private cloud for EHRs, teleconsultation, and telediagnosis services require a volume of online traffic calculated at being able to reach 2 Gbps per consultation. This may entail an average cost of €500/month.

**Conclusions:**

The security solutions put forward for each eHealth service constitute an attempt to centralize all information on the cloud, thus offering greater accessibility to medical information in the case of EHRs alongside more reliable diagnoses and treatment for telecardiology, telediagnosis, and teleconsultation services. Therefore, better health care for the rural patient can be obtained at a reasonable cost.

##  Introduction

###  Background

Taking into account the economic, social, technological, and cultural transformations attached to the term “information society”, we as citizens, professionals, and lawmakers need to reconsider our standpoint regarding the population’s health requirements. Scientific advances, improvements in diagnosis, and therapeutic measures, in addition to a healthier lifestyle, have helped to add years to the life of the population. Within this context, the emergence of telemedicine and telecare therefore becomes increasingly relevant. In modern day society, it is essential for doctors and other professionals to be able to provide their customers (patients) with the best possible information about the illness they have. Time, distance, and physical hindrances can no longer justify the barrier between the patient’s illness and the best way of combating it, regardless of where one needs to go to obtain the solution. It is in such cases where telemedicine takes on more relevance by facilitating the use of expert advice.

Telemedicine can and must play a major role in situations where urgency, geography (rural areas), and other conditions (isolation, bad weather, catastrophes) require the use of this new health care model. This is similarly required in cases where towns, villages, or regions lack minimum services due to a critical lack of staff, such as in developing countries.

However, as telemedicine evolves, the need for certain basic principles that are accepted when put into practice becomes more apparent. We can view eHealth as a form of health practice backed up by electronic processes and information and communication technologies (ICT). Telecardiology, telediagnosis, teleconsultation, and electronic health records (EHRs) therefore constitute eHealth services devoted to improving the quality of a patient’s treatment in terms of ensuring availability of specialists and reducing the need to travel, as well as being able to ensure a swift diagnosis and a second expert opinion.

The possibility of virtualizing resources on a cloud [1,2] will provide health care staff with sufficient mobility and accessibility to be able to transparently check any data they may need without having to worry about its physical location—they can still perform the tasks they deem appropriate using the information they require at any time [3-13]. Given that the data will not physically be in the same place as from where access is gained, special attention needs to be paid to how such services are requested while still ensuring optimum levels of security and privacy [14-17].

The combination of cloud computing with eHealth services can provide us with a virtual view of resources regardless of the geographic location or physical space they may occupy [6]. Fernández-Cardeñosa et al proposed two examples of cloud-based solutions for EHRs. One of them applied to a large hospital and the other one to primary care centers [18]. Although they conducted an economic analysis of the solutions, they failed to specify security aspects in the course of their work. Rodrigues et al later analyzed the security requirements of EHR solutions on the cloud [19]. They put forward secure and robust cloud-based solutions for equipping a set of rural health centers near Valladolid, Spain (where the hospital is located) with eHealth services such as EHRs, telecardiology, teleconsultation, and telediagnosis. These solutions may serve as a model when implementing eHealth services in other centers in other regions. In addition to providing models for introducing such secure solutions, we explain the importance of security within the framework of eHealth and any possible security frontiers.

In this paper, we discuss the security provided on the cloud within the framework of eHealth and its frontiers, the scenario chosen for the introduction of solutions, the authentication common to all solutions, and each eHealth solution (ie, EHRs on the cloud, telecardiology, teleconsultation, and telediagnosis).

Although the cloud offers obvious advantages, it can equally generate major concerns. When a network structure is run, it is exposed to denial-of-service attacks. If a user were able to take control of a service provider, that user would then be able to cease online services, with an ensuing cost of putting them in operation again. To put a stop to such attacks, the use of synchronized cookies as well as establishing a limit on the number of connected users help to neutralize Distributed Denial of Service (DDoS) attacks [20]. Another attack that the cloud may experience is in the case where, if the Secure Sockets Layer (SSL) is incorrectly configured, customer authentication will not be as expected, thus giving rise to a breach in security.

Therefore, the matter of security in cloud computing is a determining factor for this technology and one of the factors that can jeopardize its smooth running. How it inserts the user’s details and operates with the user’s programs—all within a physical place that does not belong to the user—may prove to be extremely discouraging for many. Well-known fraudulent methods used to obtain information such as phishing and botnets, etc, constitute serious threats to an organization’s data and software [8]. One of the greatest concerns is then how to store data on the cloud—data should be transferred and stored in coded format by using proxies and agents to isolate the customer from direct access to shared storage on the cloud. Registers, audits, and compliance with regulations are features that require planning in cloud computing systems, and the concept of presence linked to identity must also be taken into account. The Internet is shown to be a flexible yet none-too-secure network—the greater the distributed system, the greater the possibility of attacks. Cloud computing is subject to the same vulnerabilities as Internet applications, plus others that may emerge from virtualizing and sharing resources and additional subcontracted services [21,22].

To assess the risks of implementation on the cloud, we need to conduct an analysis to determine which risks may be sensitive on our cloud and which mechanisms are to be used, etc. It is highly advisable for us to have an image of security that we are able to analyze in the search for vulnerabilities and dangers, so that we when we have doubts about the system’s reliability, the only thing we need to do is return to that secure image.

Regarding security frontiers, the hardware/software bundle for the Cloud Security Alliance (CSA) model is taken as a reference for the cloud where the lowest level is Infrastructure as Software (IaaS), with Platform as Software (PaaS) and Service as Software (SaaS) above this. As levels increase in the bundle levels, each service model inherits the features of the one below—advantages and disadvantages as well as possible risks. IaaS offers the infrastructure, PaaS adds frameworks for developing applications, transactions and control structures, and SaaS is an operative environment with user applications, administration, and interface. From bottom to top, IaaS has the lowest functional and integrated security levels, and Saas the highest. Therefore, depending on which level we find ourselves, we will have certain security features or others, as well as separating the responsibilities attached to the different levels of service model according to frontiers [22,23].

In the case of the SaaS model, the supplier offers security as part of the agreement regarding level of services, in which levels of compliance, Ugovernance, and responsibility for the whole bundle are stipulated. In the case of the PaaS mode, the security model can be defined in such a way that the supplier includes the software framework and a middleware layer—with this model, the customer would be responsible for the security of the application and the user interface on the upper part of the bundle. Last, the model with the lowest level of integrated security is IaaS, in which anything involving software of any type is the customer’s problem.

###  Case Study Scenarios

**Figure 1:**
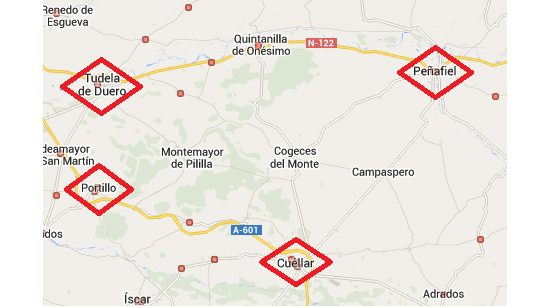
Outline of rural health centers close to the hospital in Valladolid.

The scenario proposed for offering secure eHealth solutions on the cloud is a set of rural health centers that provide different rural areas with basic care, with the most serious cases being those that require specialist care at the closest hospital—in this case, the one in Valladolid. The municipalities chosen are Peñafiel, Cuéllar, Tudela de Duero, and Portillo. The area has 36,000 inhabitants in total, each of which is covered by one doctor and one nurse who provide a physical presence. In addition, these health centers cover small neighboring villages, meaning that there is a variable flow of patients, and therefore numbers cannot be calculated exactly. These four health centers were chosen because of their proximity to the hospital in the regional capital and because they are the municipalities with the greatest number of patients referred to the cardiology department of the specific hospital (around 8% of hospital referrals). By way of an example of a rural health center, the town of Peñafiel in the province of Valladolid, Spain, was chosen where 5677 patients were seen in 2013 with an average 473.08 patients a month and 15.6 a day. Most cases (93%) were dealt with at the health center itself, while 6.8% were referred to the hospital because they were unable to be dealt with properly at the rural health center. Figure 1 shows the chosen health centers in terms of proximity to the regional capital where the hospital is located (ie, Valladolid, Spain).

Although the rural health centers chosen depend on the municipality to whom they provide the service, our choice is based on the premise that they are an acceptable size. The fact that they are centers that lack specialists and/or means available for more complex treatment should also be taken into account. Cardiology cases are referred to the relevant hospital as such cases are not dealt with onsite, as well as other cases of different types.

Therefore, special use of EHRs is made at rural health centers, where the doctor dealing with the consultation modifies the details of the patient being seen. Some patients are then sent on to hospital. The flow of patients at the rural health centers has to taken into account in this study. It is important to note that there are rural health centers with more than one doctor and nurse on duty and even a pediatric and physiotherapy unit. Moreover, the number of patients attended to may vary, reaching up to 400 or 500 a day during doctors’ surgery hours, which might be the case with the health centers in Peñafiel and Cuéllar.

Special attention needs to be paid to a swift cloud computing solution for all services, as most of the information being treated will simply be text, meaning that such a flow of data has to be optimized to ensure that electronic consultation and modifications are carried out with as little delay as possible.

Another issue to consider is the fact that the only professional who is authorized to modify EHR information is the doctor, as other medical staff have no access to such information. This is a major factor when consulting the patient’s information so as to ensure the privacy of the patients being seen. The doctor is also the only one able to attend to teleconsultation and telediagnoses.

The means and technology exist in these four health centers to implement service providers in each center, along with the infrastructure needed to ensure good quality communication. All four centers have broadband Internet. The parameters for choosing a secure infrastructure on the cloud are the cost per unit of time and online traffic.

##  Methods

iCanCloud [24] software is used to carry out the simulations within the proposed scenario, which provides a type of modeled physical structure that the user can work with. Using this tool significantly reduces the cost of contracting resources because the user can perform a range of simulations of their future infrastructure until they achieve an efficient result in terms of cost/unit of time. We note here that some of the data from each health center are approximate when choosing the infrastructure required to provide access to each eHealth service (eg, regarding the volume of data being handled in each center). This information is required when offering an infrastructure to the EHR system. For telediagnosis and teleconsultation services, the most important criteria when selecting the technology are quality of the image and availability of the system, in real time, as in the case of a telecardiology solution. A secure system has been chosen for all solutions that provides access to the information handled (both text and images), which can be accessed via authentication on the part of the user—both patient and doctor.

##  Results

### Results

Here we describe and compare the key elements associated with eHealth services offered via the cloud and the infrastructures required in terms of security for each. The first stage is common to all services with respect to secure authentication by patients and doctors.

###  Secure Authentication for All Services

**Figure 2:**
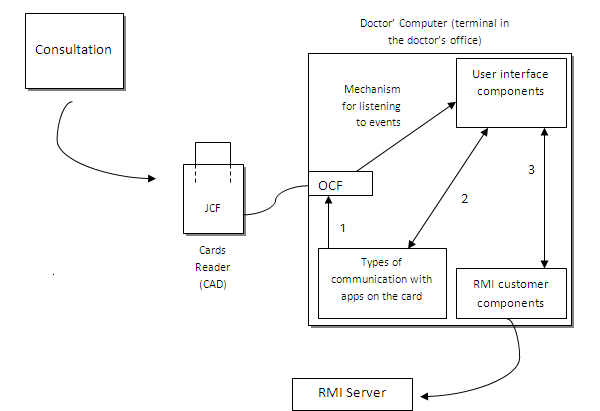
Sample consultation showing operation of smartcards.

Regardless of the type of eHealth application that needs to be implemented together with the cloud computing technology, all solutions must be correctly authenticated. This is a fundamental, indisputable factor that needs to be covered, as otherwise data might be used by unauthorized persons, which would entail loss of information, fraudulent use, etc, which is unacceptable for this type of an environment. In all cases, a private cloud has been chosen in order to increase security measures. Therefore, diverse encoding technologies, digital signatures, tools, and technologies have been researched to offer an effective, viable solution for correct authentication by health professionals. The decision has also been made to implement the use of smartcards in the system as a feasible and efficient solution for maintaining identity with a suitable degree of security for this joint cloud computing system for any eHealth application introduced on the cloud. Figure 2 shows an example of a consultation in which operation of the doctor’s computer and the CAD card reader are observed in detail.

The computer(s) located in the professional health staff’s rooms are equipped with a Card Acceptance Device (CAD), which is a smartcard reading device that takes charge of the customer part of the system. This is sufficient for a Java Virtual Machine (JVM) and an OpenCard Framework bookstore (OCF). The OpenCard Framework or OCF of a smartcard is simply middleware implemented in Java that enables an application to be aware of the card’s presence and to interact with it in accordance with ISO/IEC standards 7816-4, -8, and -9 [25]. All the aforementioned interact jointly with health care software from the system in each terminal where we have the CAD installed.

OCF is developed by OpenCard Consortium, including IBM and other leading firms in the sector. The framework is implemented in Java and provides an environment that can be developed independently of the card manufacturer. In terms of architecture, OCF is located between the CAD and the host application in the computer. It is hoped that OCF will be integrated in smartcards destined for use in health care systems [26].

The customer smartcard package that is run in the customer equipment ensures communicates both for the doctor’s card and for the patient’s. It prepares the environment for card communication with the computer via the Application Protocol Data Unit (APDU) protocol, that is, the ISO 7816 smartcard communication protocol. This package is designed as an API to provide what we might refer to as summaries of customer cards, including APDU communication interfaces. In this way, the components of the user interface are able to use objects provided by this package without having to continually struggle with conversations about smartcard data structure.

An object known as the Card Manager take charges of initializing and shutting down the computer, and establishing a secure communication channel between the smartcard and the session that happens to be open at a specific time. The data regarding sessions are handled by objects known as DoctorSession and PatientSession, which communicate with each other as explained via APDU.

The smartcard terminals act as customers in the remote method invocation (RMI) protocol by calling remote object methods. As previously mentioned, the customer equipment does not contain any software in charge of accessing databases and making enquiries, meaning that the customer software only contains the components of the user interface (eg, types of Java) and ways of displaying layers of Model View Controller (MVC) architecture.

###  Electronic Health Record Service: Infrastructure and Features

The aim is to provide support to an EHR application via cloud computing within the rural health center environment. A series of aspects need to be taken into account prior to considering any solution or implementation. Specifically, the fact that the solution should be secure and robust must be taken into account, and privacy must be provided via efficient authentication support given to ensure that EHRs can be accessed by authorized health care staff. The storage system needs to be scalable yet fair. There is a small number of health records to be dealt with, according to the rural population registered at the center. The data must be replicated, as it is of a sensitive nature, and this process will be repeated throughout the pairs of application scenarios. It is vital that the information be backed up against any possible fraudulent use that may give rise to loss, or in case the information itself becomes mislaid. The quality of communication needs to be of acceptable standard, and there should also be a fiber service to prevent any delays in communication as far as possible. We will likely experience a certain network overload when making simultaneous requests for access to EHRs, meaning that a decent network infrastructure will not manage to avoid everything, although it will do so to a large extent. Each session involving access to data needs to be assured in detail, from access to the session to shutting it down—which must be secure and never exposed to any possible fraudulent use of the information. Although there may be major information loads in terms of movement, EHRs are mostly written in flat text. Despite the fact that it may sometimes include images from specialists, it is assumed that data traffic will be at an average level. This will affect the structure of the proposed network and, as a result, the storage system.

A general outline of the solution is as follows. The first essential step is access to the cloud by the doctor or professional health care worker. In the case of the solution, access to data can be gained only if the doctor is correctly authenticated in the system, otherwise no secure connection can be established with the information system where the EHRs are located.

**Figure 3:**
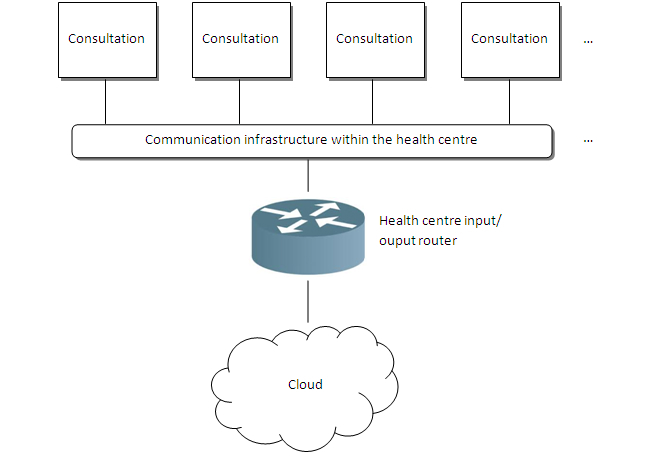
Connection outline for each rural center.

**Figure 4:**
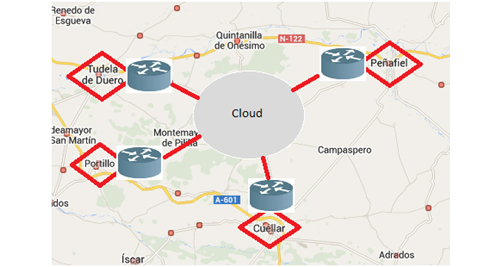
General connection outline for the rural health centers with the cloud.

The general connection outline within each rural center is shown in Figure 3. Research has been carried out into offering a secure solution using cloud computing technology—an eHealth application fully implemented on the cloud together with the elements required for security and storage—so as to supply the service with EHRs on the cloud for rural health centers. Figure 4 shows the ideal implementation.

**Figure 5:**
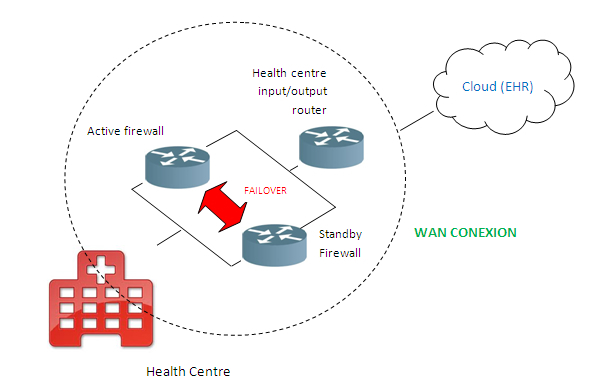
General connection outline for each rural health center.

The outline of each health center to be connected to the cloud is represented in Figure 5. Each data input and output from and to the cloud passes through two Cisco firewalls with Failover IP configuration in order to obtain a secure and robust service 24/7.

We will subsequently find the input and output router for each rural health center to the cloud. For the solution involving implementation of EHRs, we decided to always install load balancers in communication together with several Internet service providers, due to the fact that we are unaware of the exact amount of information to be received.

**Figure 6:**
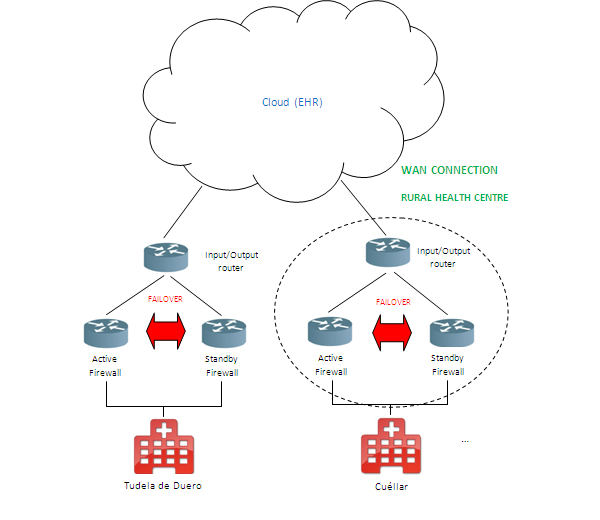
General connection outline for health centers with the cloud.

Each center will have two Cisco firewalls in Failover IP configuration so as to provide the system with a secure and uninterrupted service. This ensures secure access to the system in the cloud and a shielded communication system between the health centers and the storage system on the cloud. The communication channel is assured via the input/output router of each health center with the cloud. Figure 6 shows the outline for implementation of the EHR system. These elements will have been used previously in other solutions as well as the infrastructure required for each consultation, to enable the smartcard to be used by doctors and the local area network (LAN) infrastructure to be established at the center for the output of data to the outside.

**Figure 7:**
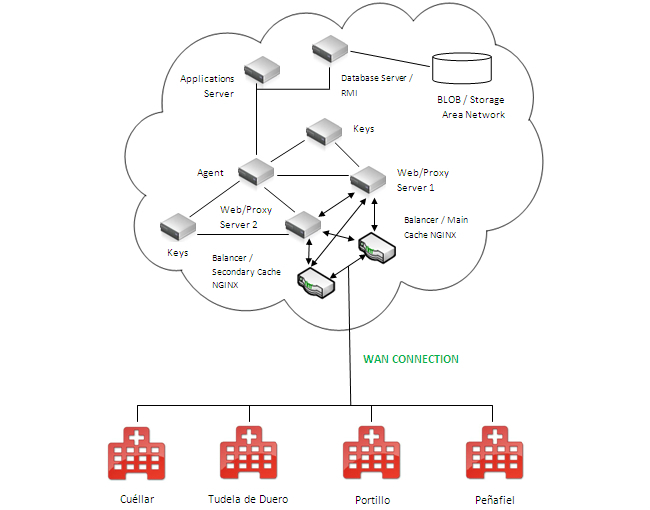
Outline of the infrastructure on the cloud for full implementation of EHRs.

In Figure 7, the infrastructure on the cloud for the full implementation of EHR system is shown. A scalable solution is proposed in terms of capacity for information, which is secure thanks to the shielded communication channels created. Privacy is also granted via use of smartcards exclusively held by authorized health care staff from the center. The cost per month of this solution is around €500.

A LAN connected to the communication output elements will be available at any of the health centers, whereby each doctor may use their smartcard in the CAD via each consultation to obtain the precise information they need on each patient. The problems associated with simultaneous requests from various health centers are eliminated thanks to the load balancers and the use of several Internet service providers who will show the result to the doctor when they require it. The request for information about health records will be dealt with by the database service provider, which understands that there is a staff member authorized to be authenticated using the smartcard system, meaning that the information shown by the Internet service provider can be displayed by the doctor.

###  Telecardiology Service: Infrastructure and Features

The differences between the telecardiology service that has been fully implemented on the cloud in rural health centers and implementation of the previous eHealth application on the cloud is explained in this section.

Those features that are similar to full implementation of EHRs on the cloud are as follows: the wide area network (WAN) connection and rural health center infrastructure, and secure authentication using smartcards. Storage retains the same infrastructure and filing systems, as well as access to them and the StorageGRID technology to administer them. The database and Internet service providers perform the same tasks together with the agent, and the load balancers will remain essential for this solution.

The differences with EHR implementation are as follows: there must be communication with the hospital in order to obtain telecardiology support. As we are dealing with a rural environment, this is essential given that the rural health center does not provide this service. A new LAN infrastructure is needed, if one did not previously exist, to provide connection support for electrocardiograms, echocardiograms, Holters, and angiocardiographs (ie, all the electronic tools required for cardiology diagnosis on the communication side of the hospital). A cardiology connection infrastructure is required at the closest hospital with the cloud so as to provide telecardiology support for the rural health centers.

Load balancers and several Internet service providers will be used to provide support for all the requests entering the system on the cloud, as this could be a large number due to the unevenly distributed population within the municipalities. The filing system and also the security methods are as in the previous models; therefore, the cost per month of such a solution is around €450.

In this scenario, we decided that the health centers should have direct contact with the hospital via the cloud. In this way, doctors from the rural health centers may upload data onto the cloud and the specialist in turn may upload images they as they see fit onto the filing system. They can also leave messages for the doctor or consult the doctor from the rural center for diagnosis of some specific case regarding the patient. We also decided to introduce this system to ensure that the telecardiology application goes directly to the cardiologist at the hospital, so that they will be able to offer better quality treatment to each specific patient with direct care provided from the hospital. Using electronic tools provided by cardiologists from the hospital, the image can then be created locally and subsequently uploaded onto the cloud. Once the application service provider has finished processing the image, it records it in the database and returns the result to the Internet service provider so that the specialist or general practitioner may display this result and make the relevant decisions in each given case.

The following are used: secure information channels using firewalls and the proxy/Internet-agent with respective coded keys, the BLOB + SAN (RAID 0+1) filing system with back-up copy administered using StorageGRID, which is able to make back-up copies in different physical devices, and smartcards for authentication of professional health staff. In this way, a fully shielded solution is obtained for using patient data both on the communication side of the health center and by the specialist at the hospital (if required).

Regardless of where the health center is located, the cloud is always available for attending to a cardiology service from the closest hospital. Thanks to the BLOB, the data service provider will be unburdened in attending to requests from different centers without overload problems. Similarly, the application/transaction service provider provides an optimum flow of data because it uses a message queuing system to create a solid flow that it is able to process.

###  Teleconsultation and Telediagnosis Service: Infrastructure and Features

Teleconsultation and telediagnosis are services with a major flow of traffic—an important factor when seeking a suitable cloud computing solution. The infrastructure itself on the cloud must be prepared to support a major information traffic load, as we start from the premise that the hospital infrastructure is prepared to support major loads of this type as in previous cases.

Doctors may consult specialists and specialists may consult other specialists or there may be a variety of combinations that require a tolerant system with a properly structured major work load to be implemented.

These are the two costliest eHealth services due to the fact that they have a major influence over the rest. This leads to the search for a less costly solution, whereby a sufficient cloud computing scenario is proposed that can at the same time be adapted as much as possible without including an excessive number of elements that could be detrimental by giving rise to more traffic, as well as raising the cost of the infrastructure.

The solution adapts perfectly to the previous case in which the specialist could be consulted and images uploaded by the cardiologist, etc. We consider the same infrastructure as in the previous case since consultation and diagnosis may be required of specialists by the hospital. This decision was made for different reasons, as load balancers and Internet service providers would be needed for a possibly major flow of traffic from different rural health centers. Diagnosis or consultation by specialists located at the hospital may prove to be necessary. Therefore, we decided to implement the same infrastructure as in the previous case so as to be able to maintain communication with the hospital and for specialists to upload images, send messages to doctors, respond to inquiries, and help with diagnoses, etc. This will help improve the quality of the patient’s treatment.

The doctor may access the patient’s data in the same way as in the previous case, by modifying, displaying, or updating information about the latter via the use of smartcards by authorized professional health care staff—in this specific case, the doctors at the rural health center.

The solution to this scenario is adapted to the requirements offered by teleconsultation or telediagnosis. As the information is always made available on the cloud via online portals, there is open communication with specialists and support can be provided regardless of location. This translates into a major cost in infrastructure for the health care body and an improvement in the patient’s quality of life.

Now that each of the secure solutions for the different eHealth services has been described, Table 1 shows a comparison between the three solutions according to criteria such as type of service, type of cloud, estimated cost per unit of time, estimated amount of traffic on the network, and key security elements.

**Table 1:** Comparison between cloud-based solutions for the different eHealth services.

Type of service	Type of cloud	Estimated cost (€/month)	Traffic on the network (Gbps)	Key security elements
EHRs	Private	500	1	Firewalls
				Load balancers
				Smartcards
				Filing system
Telecardiology	Private	450	1	Firewalls
				Load balancers
				Smartcards
Teleconsultation/ Telediagnosis	Private	500	2	Firewalls
				Load balancers
				Smartcards

## Discussion

### Principal Considerations

After researching cloud computing technology to discover whether secure solutions can be offered to telecardiology, teleconsultation, telediagnosis, and EHR eHealth services on the cloud for different rural health centers, we have offered an optimum infrastructure as a viable proposal for each given case. Access to these services on the cloud enables more reliable treatment and diagnosis to be offered, above all in environments that lack certain services such as health centers in small municipalities where no medical specialists exist.

We opted for a private cloud for all services in order to further ensure security levels. The costs of the infrastructure network at the four health centers are estimated, with the costliest being those assigned to the EHR and teleconsultation and telediagnosis service. As far as the estimated amount of network traffic is concerned, the service that gives rise to the most traffic is that of diagnosis, owing to the images and videos sent via the network.

The use of computing technology on the cloud alongside the proposed smartcard system for doctors’ authentication helps health centers to electronically manage all health-related data about patients and enables them to make any modifications in a reliable manner—meaning that privacy, security, and robustness are assured in an extremely sensitive data system.

Any authorized staff member, doctor, or other health care professional may access the services provided by the different eHealth applications at any time and from any location within the different scenarios proposed, under the assurance that their privileges be maintained on the cloud by using their smartcard to access data.

Control of access to the system, and the use of Cisco firewalls configured using the Failover IP, greatly enhance security offered by the use of coded keys via proxy and the agent. The storage system proposed enables back-up copies to be created on physically independent elements on the cloud. Together, they increase the privacy and security of all communications from start to finish, as well as ensuring the robustness of the data.

### Conclusions

The research carried out for this paper on cloud computing technology, in addition to other technologies required to provide excellent authentication of the system, has allowed us to suggest a solution adapted to each eHealth service at rural health centers that offers security, privacy, and robustness and can also be deemed optimum for a large number of requests on the cloud.

Within a common scenario of cloud configuration, a customer is initially unaware of the requirements they need to provide infrastructure to the software housed, and even less so at any optimum level. One of the future areas of research would be to analyze the eHealth services proposed in this paper in other scenarios such as hospitals or an individual health center.

## Abbreviations

APDU: Application Protocol Data Unit
CAD: Card Acceptance Device
CSA: Cloud Security Alliance
DDoS: Distributed Denial of Service
EHR: electronic health record
IaaS: Infrastructure as a Service
ICT: Information and Communications Technologies
JVM: Java Virtual Machine
LAN: Local Area Network
MVC: Model View Controller
OCF: Open Card Framework
PaaS: Platform as a Service
QoS: Quality of Service
SaaS: Software as a Service
SSL: Secure Sockets Layer
WAN: Wide Area Network
